# The College of Nursing Coat of Arms and Badge

**Published:** 1919-12-13

**Authors:** 


### The College of Nursing Coat of Arms and Badge.

### THE COMPETITIVE DESIGNS.

LAST August the Council of the College of Nursing announced that it proposed to apply for a coat of arms and a badge, and that members of the College and all interested in it were invited to submit designs for (*a*) a coat of arms and (*b*) a badge, with a view to these being submitted, if thought suitable, to the proper authorities. THE HOSPITAL offered a prize of £10 for the best design for the coat of arms, and the College offered a first prize of £7 10s. and a second prize of £2 10s. for the best design for a badge. Some ninety-six designs were submitted for the badge, and the Council has decided to award the first prize of £7 10s. for this design to Miss I. Neville, 10 St. Mary's Road, Leamington, and the second prize of £2 10s. to Miss G. Peers, 82 Upper Gloucester Place, Dorset Square, N.W. 1. Fifty-five competitive designs for a coat of arms were received. It was felt that the coat of arms should bear some relation to the College or its objects, and whatever design might be chosen that it should be symbolic in character. The Committee appointed by the College Council to consider the designs reports that no design fulfils this condition, and in the result the Committee of Selection have made no award.

